# PARTNERING WITH COMMUNITY ON A SUCCESSFUL FEDERAL FUNDING APPLICATION FOR MESOTHELIOMA CAREGIVERS

**DOI:** 10.1093/geroni/igae098.3878

**Published:** 2024-12-31

**Authors:** Amanda Woodward, Maria Towe, Jen Hirsch, Julie White

**Affiliations:** Michigan State University, East Lansing, Michigan, United States; Mesothelioma Applied Research Foundation, Washington, DC, District of Columbia, United States; Michigan State University, East Lansing, Michigan, United States; Mesothelioma Applied Research Foundation, Washington, District of Columbia, United States

## Abstract

The Michigan State University School of Social Work (MSUSSW) and the Mesothelioma Applied Research Foundation (Meso Foundation) collaborated on a successful application for federal funding to develop and conduct feasibility testing of an online caregiver support intervention. The process of developing the proposal and the design of the four-year project follow key principles of the Community-Engaged Research Framework described by NORC at the University of Chicago. The research questions emerged from a survey the Meso Foundation administered to its community, thus aligned with a foundational component of community engaged research where the community drives the research focus. The team includes members with lived experiences whose time and expertise are incorporated through processes that promote shared decision making (e.g., co-principal investigators) and budgeted compensation for their work. Information about research processes, resources, and timelines are openly shared with all team members through password-protected cloud-based storage. Meeting schedules, modes of communication, workplans, and decision-making protocols are established collaboratively and revisited regularly throughout the work. The project itself engages patients, caregivers, those who have lost a loved one, and health professionals through in-depth interviews and focus groups to better understand the caregiver experience from diagnosis through end of life. Participant experiences and feedback will be used to develop and refine an online caregiver support intervention. The feasibility of the intervention will then be tested in an intervention-only design. This poster will describe the six key principles of community-engaged research and the ways we are implementing them in more detail.

